# The Durability Assessment Methodology of Power Engineering Equipment Under Thermo-Mechanical Fatigue Using the Example of the HR6W Alloy

**DOI:** 10.3390/ma19050891

**Published:** 2026-02-27

**Authors:** Michał Paduchowicz, Tomasz Dobosz, Artur Górski

**Affiliations:** Faculty of Mechanical Engineering, Department of Machine and Vehicle Design and Research, Wrocław University of Science and Technology, 50-370 Wrocław, Poland; tomasz.dobosz@pwr.edu.pl (T.D.); artur.gorski@pwr.edu.pl (A.G.)

**Keywords:** high-alloy materials, computer methods in mechanics, Coffin–Manson and Ostergren parameters

## Abstract

This article presents an innovative methodology for assessing the durability of power engineering components under thermo-mechanical fatigue conditions. The approach integrates laboratory low-cycle fatigue tests of alloy specimens at elevated temperatures, measurements of working-medium parameters obtained from operating industrial equipment, and numerical simulations performed using the finite element method. Durability is estimated on the basis of curves describing the relationships between critical parameters such as the Coffin–Manson and Ostergren parameters and the number of cycles to failure. Within the region of the structure identified as the most susceptible to fatigue damage, the orientation of the critical plane is determined with respect to the corresponding criterion functions. This allows the calculated criterion values to be correlated with experimental data, enabling the determination of the incremental durability loss of the component. The proposed methodology is distinguished by its practical applicability and the possibility of incorporating both proprietary fatigue test results and data reported in the literature.

## 1. Introduction

Due to the continuously increasing demand for electric power, ensuring the reliability and continuity of electricity supply has become a matter of critical importance. This, in turn, requires maintaining an adequate technical condition of the engineering systems involved in power generation. In conventional coal-fired power units, whether using hard coal or lignite, the components that largely determine the availability of the entire installation are the thick-walled elements, including the boiler superheater chambers, as well as the valves and casings of steam turbines. These components are exposed to working media characterised by high temperatures, pressures, and mass flow rates.

The failure processes affecting such equipment can be grouped according to the following mechanisms: high-temperature creep, fatigue, low-cycle fatigue at elevated temperatures, thermal fatigue, and thermo-mechanical fatigue.

This article focuses specifically on assessing the impact of thermo-mechanical fatigue on the overall fatigue life of components operating at elevated temperatures. Currently, several methods for assessing the durability of components subjected to low-cycle fatigue are described in the available literature. These include, among others, the method presented in [1]. The following article proposes a proprietary methodology for predicting life loss resulting from thermo-mechanical fatigue. This methodology integrates laboratory fatigue testing of metal alloys at elevated temperatures, measurements of actual operating parameters of the working fluid flowing through the analysed devices, and numerical calculations using the finite element method. One of the main assumptions of the proposed methodology is the ability to use low-cycle fatigue test results at elevated temperatures instead of thermo-mechanical fatigue results. This approach has been presented in numerous scientific publications, including [2,3,4]. The above studies, similar to the methodology presented here, assume (supported by research and described in these publications) that the sum of mechanical deformation resulting from simultaneous changes in medium temperature and mechanical loading (e.g., due to fluctuations in working fluid pressure) during actual facility operation is no greater than the range of total deformation assumed during low-cycle fatigue testing at elevated temperatures. This approach also stems from the fact that during actual operation of power plants, the number of possible combinations of simultaneous temperature changes and mechanical loading is so large that it would be very difficult to reflect this in laboratory testing of samples. Furthermore, it is worth noting that conducting low-cycle fatigue testing at elevated temperatures is significantly easier than for thermo-mechanical fatigue, making it easier to obtain results that can then be used in engineering practice for durability assessment. Furthermore, the methodology allows for the simultaneous use of both data from internal fatigue tests and results published in external sources, including publications and relevant standards, which can increase its accuracy. An additional advantage of this methodology is the ability to generate model cyclic hardening curves for temperatures for which fatigue tests have not been conducted, using extrapolation and interpolation of known data from our own studies and those available in the literature.

## 2. Methodology for Estimating Durability Under Low-Cycle Fatigue at Elevated Temperatures

**Figure 1:**
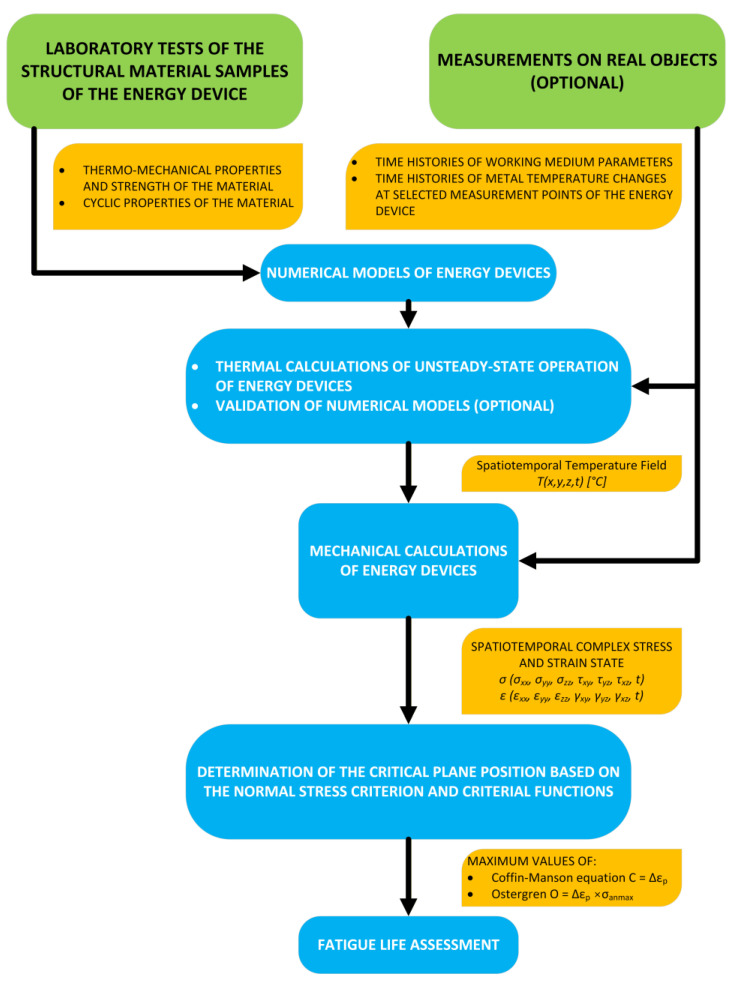
Block diagram of the proposed methodology for assessing the durability of power engineering components under thermo-mechanical fatigue.

A novel methodology has been developed for evaluating the durability of power engineering components operating under thermo-mechanical fatigue conditions (Figure 1). The approach is founded on low-cycle fatigue testing of alloy specimens designed for high-temperature applications, in conjunction with measurements obtained from operating industrial installations and numerical simulations performed using the finite element method.

This methodology does not replace TMF with LCF. When the combined mechanical and thermal strains occurring during the operation of power equipment under TMF are significantly smaller than the strains for the LCF specimen tests, this algorithm conducts LCF tests to obtain cyclic hardening curves, which then serve as input data for numerical models of material hardening. The subsequent numerical analysis and the resulting durability assessment are related to the fatigue assessment of TMF.

The first stage of the methodology involves conducting laboratory tests, including uniaxial tensile tests and low-cycle fatigue tests of specimens at elevated temperatures, in order to determine the material’s strength and cyclic properties (the monotonic tensile curve and the cyclic hardening curve). In parallel, measurements are carried out on operating industrial equipment to determine the actual thermal and mechanical loads associated with the working-medium flowing through the analysed components (e.g., steam pressure and temperature). The situation is quite different when the above methodology is applied in the design of new power units where the curves of the working medium parameters, such as pressure, temperature, and mass flow, are unknown. In such a situation, these curves should be designed based on the design and operational assumptions of the new unit and based on analogy to curves derived from actual power units with parameters similar to those of the new power boiler.

The next step in the proposed algorithm is the development of numerical models required for subsequent finite element analyses. At this stage, in addition to generating an appropriate finite element mesh, it is essential to define a suitable material hardening model that incorporates both the monotonic tensile curve and the cyclic hardening curve obtained from laboratory testing at a specified temperature. A significant advantage of the methodology at this stage is the ability to generate missing model cyclic hardening curves for intermediate temperatures.

The subsequent stage consists of performing thermo-mechanical analyses of the investigated component using the finite element method. Boundary conditions are derived from measurements of the working-medium parameters recorded on the actual installation. The calculations yield the combined stress and strain state of the analysed component. Based on these results, the region of the structure that is most critical from the fatigue-strength perspective is identified, representing the next step of the methodology.

Within this critical location, the orientation of the critical plane is determined using the normal-stress criterion together with the Coffin–Manson and Ostergren parameters. This step is essential for assessing the residual durability of the component on the basis of predefined criterion functions that relate the number of cycles to failure to specific values of the Coffin–Manson and Ostergren parameters. This allows the unit durability loss associated with a single characteristic load cycle to be quantified. Having identified the number of occurrences of this load cycle, and by performing analogous analyses for other load types, the total durability reduction in the component due to thermo-mechanical fatigue can be determined (Figure 1).

The methodology for assessing the durability of power engineering components under thermo-mechanical fatigue conditions, as presented in this article, can be applied both in the design of new installations and in the evaluation of components already in service.

## 3. Example Application of the Methodology for Assessing the Durability of Power Engineering Components

The proposed methodology is suitable both for the design of new power engineering components and for the evaluation of equipment already in service.

**Figure 2:**
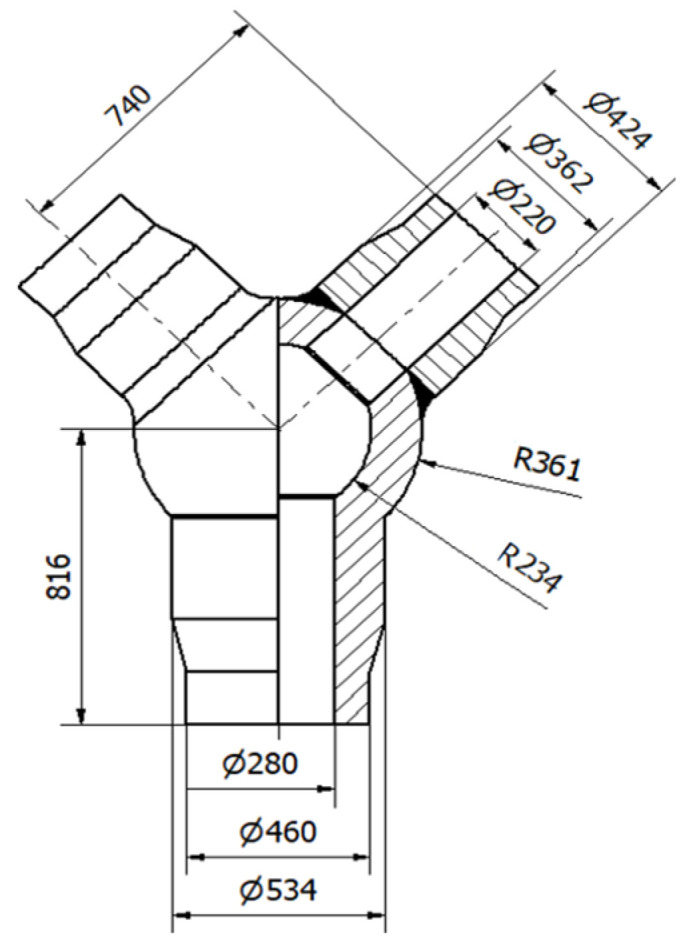
Proposed design of a forked tee made of HR6W material according to the referenced publication.

Accordingly, the methodology will be illustrated using the example of a fresh-steam pipeline tee made of HR6W alloy, designed by the authors of publication [2] (Figure 2). It is assumed that the analysed tee will operate as a component of ultra-supercritical boilers, where the maximum temperature of the primary superheated steam is 650 °C and the corresponding pressure is 30.3 MPa.

As part of the presented procedure, strength and fatigue tests of the HR6W alloy conducted at 650 °C were utilised. This nickel-based alloy was selected based on its superior mechanical performance at elevated temperatures, as it is regarded as a candidate structural material for critical components of power units operating under supercritical steam parameters.

**Figure 3:**
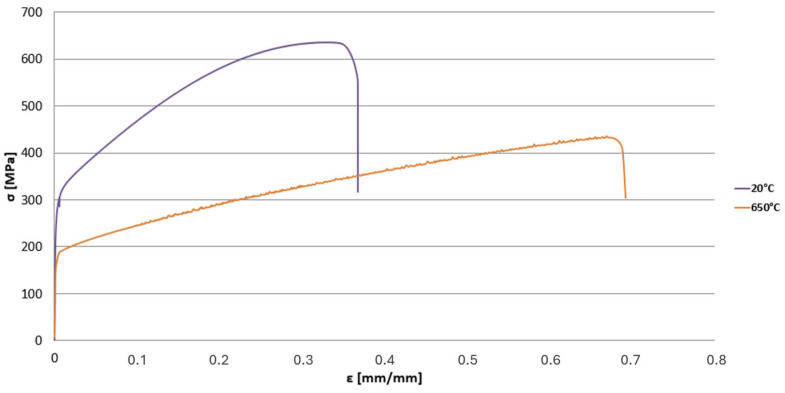
Comparison of monotonic tensile curves for HR6W steel at 20 °C and 650 °C, developed on the basis of averaged results obtained from uniaxial tensile tests performed on five specimens for each temperature in accordance with ISO 6892-2:2018 [5].

To determine the material properties required for developing the material model, a monotonic tensile test was performed on this alloy in accordance with the ISO 6892-2:2018 standard [5]. The tests were carried out at two metal temperature levels: room temperature (22 °C) and an elevated temperature of 650 °C ( Figure 3).

The next stage involves conducting thermo-mechanical fatigue tests of the selected alloy in order to obtain its cyclic fatigue characteristics. However, because such tests are time-consuming and costly, low-cycle fatigue (LCF) tests of the HR6W alloy at 650 °C were performed instead. These tests were carried out for several levels of total strain range ∆ε_C_, specifically 0.6%, 0.7%, 0.8%, 0.9%, 1.0%, and 1.2%.

The rationale for replacing thermo-mechanical fatigue testing with elevated-temperature low-cycle fatigue testing is the assumption that, for sufficiently high total strain ranges, LCF tests can reproduce the complex stress–strain state that develops in a specimen subjected to thermo-mechanical fatigue. This simplification has been presented and justified in the relevant literature, for example, in publication [6].

**Figure 4:**
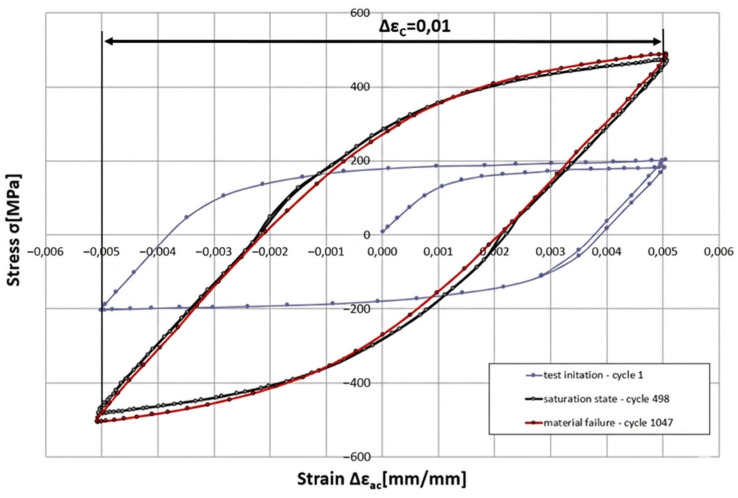
Example hysteresis loop obtained in a low-cycle fatigue test for a total strain range of ∆ε_C_ = 1.0% at a temperature of 650 °C.

Figure 4 presents an example hysteresis loop obtained for a total strain range ∆ε_C_ of 1.0.

**Figure 5:**
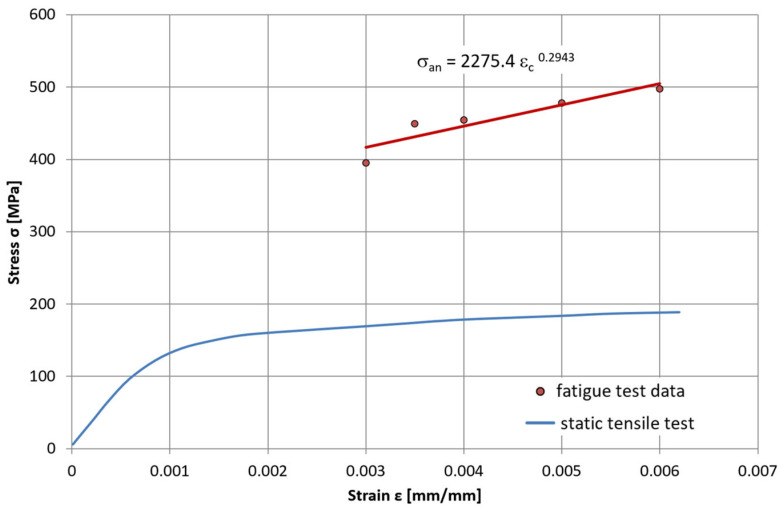
Cyclic hardening curve determined at an elevated temperature of 650 °C, compared with the curve obtained from the monotonic tensile test of the specimen.

At the same time, an increase in the maximum stress value within the hysteresis loops (the loop apex) is observed for the saturation states corresponding to different levels of total strain. This indicates that HR6W steel undergoes cyclic hardening at 650 °C as a result of repeated deformation. This behaviour was characterised using the cyclic hardening curve shown in Figure 5, which was derived from the hysteresis loops corresponding to the remaining total strain ranges ∆ε_C_.

**Figure 6:**
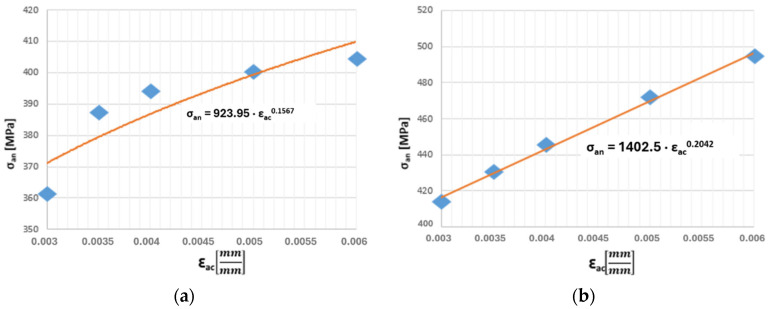
Cyclic hardening curve for the HR6W alloy determined based on literature source [4]: (**a**) at 20 °C, (**b**) at 600 °C.

Taking into account the obtained results in the context of their further application, particularly in numerical strength calculations, a major limitation may be the insufficient number of cyclic hardening curves. Characteristics for intermediate temperatures up to 650 °C are missing. Therefore, a method for obtaining these curves was developed. First, in the case of the HR6W material, the study reported in Ref. [7] was used for this purpose. From this publication, two additional cyclic hardening curves were obtained for temperatures of 20 °C and 600 °C (Figure 6a,b).

The methodology for assessing the durability of power engineering equipment presented in this study is, in its fundamental assumptions, based on broadly understood modelling. Consequently, as a next step, the cyclic hardening curves, both those obtained from the authors’ own experimental investigations and those acquired from the literature, were used to mathematically determine the cyclic hardening curves for the remaining temperatures of the HR6W material, i.e., in the range between 20 °C and 600 °C.

According to the literature source [8], the cyclic hardening curves take a form that can be described by the following relationship:

(1) $$\sigma_{an}=k\cdot {\varepsilon_{ac}}^{n}$$

where

- k—fatigue strength coefficient for the cyclic strain curve;
- n—cyclic strain hardening exponent for the cyclic strain curve.

Table 1 presents the values of the coefficients k and n for the cyclic hardening curves corresponding to three temperatures: 20 °C, 600 °C (literature data), and 650 °C (experimental data). These values were determined based on our own research described in publication [9] and in an article by other authors [2]. There are no generally available values for the remaining temperatures.

**Figure 7:**
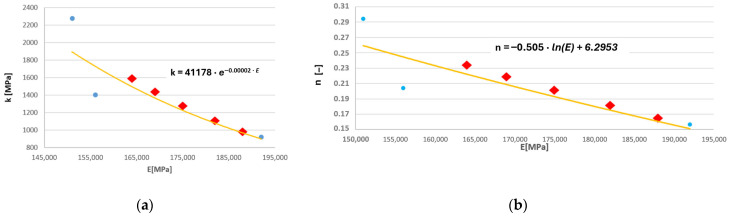
Relationships between (**a**) parameter k [MPa] and Young’s modulus, and (**b**) parameter n [-] and Young’s modulus.

In order to improve the accuracy of the calculations, an attempt was made to determine cyclic hardening curves for intermediate temperatures. For this purpose, based on the data summarised in Table 1, plots were constructed showing the dependence of the coefficients k and n on the Young’s modulus corresponding to specific temperature values (Figure 7a,b). The Young’s modulus was selected as the independent variable for these functions because it influences the stress amplitude σ_an_ and the total strain amplitude ε_ac_, i.e., the parameters appearing in relation (1). Consequently, the shape of the cyclic hardening curve depends indirectly on the Young’s modulus.

In the above figures, the red markers indicate the values of the coefficients k and n determined on the basis of the proposed relationships obtained through linear regression using the data listed in Table 1 (blue points in Figure 7a,b). The exact values of these coefficients, both the actual ones, i.e., derived from the authors’ own experimental results and literature data, as well as the model values calculated using the defined mathematical relationships, are presented in Table 2. These values are listed together with the corresponding Young’s modulus values for the respective temperatures. The dependence of the Young’s modulus of the HR6W alloy on temperature was defined based on data from the publication [2] (for temperature values of 100–600 °C) and our own research consisting of carrying out a static tensile test of samples at room temperature of 20 °C and an elevated temperature of 650 °C (Figure 3).

**Figure 8:**
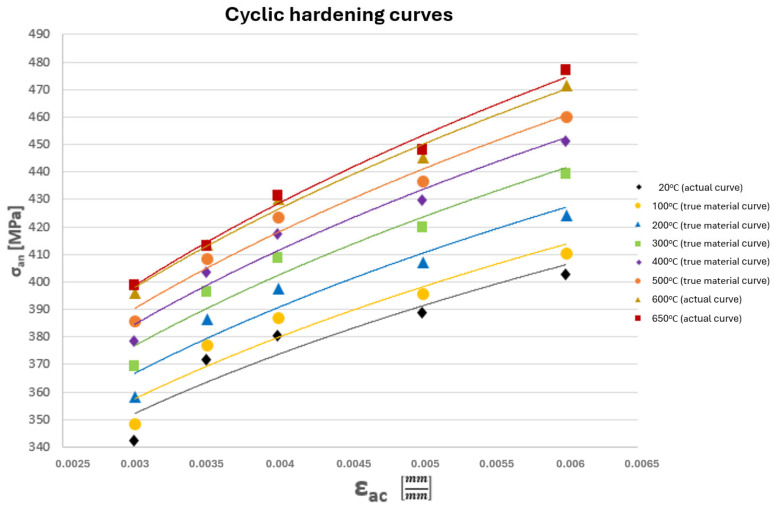
Cyclic hardening curves of the HR6W alloy obtained from laboratory test results on specimens (experimental curves) and approximated using mathematical relationships (model curves).

Based on the coefficient values listed in Table 2, model cyclic hardening curves were determined for the remaining temperatures (Figure 8).

One way to verify the accuracy of the obtained k and n coefficients is to visually verify the generated model cyclic hardening curves. This involves the shape of these curves (sufficient similarity to the curves obtained from tests) and their spacing (evenness of their position relative to each other). Taking into account the above criteria, the obtained model cyclic hardening curves for the HR6W alloy at temperatures of 100–500 °C are correct.

**Figure 9:**
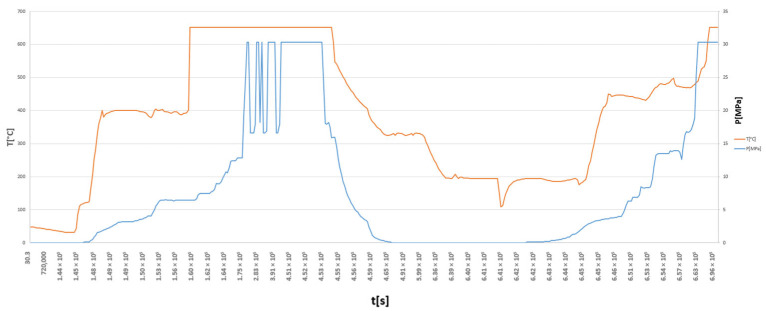
Adopted time-dependent variations in pressure and temperature of fresh steam flowing through the tee of the pipeline made of HR6W material for the reference computational cycle.

Subsequently, the operating characteristics of the analysed HR6W alloy tee were determined. This material is not currently used in any components of existing power-generation units. Therefore, the time histories of the working-medium parameters for the analysed pipeline tee were assumed based on similarity to the pressure, temperature, and mass flow rate curves observed in other units operating under comparable conditions (Figure 9).

**Figure 10:**
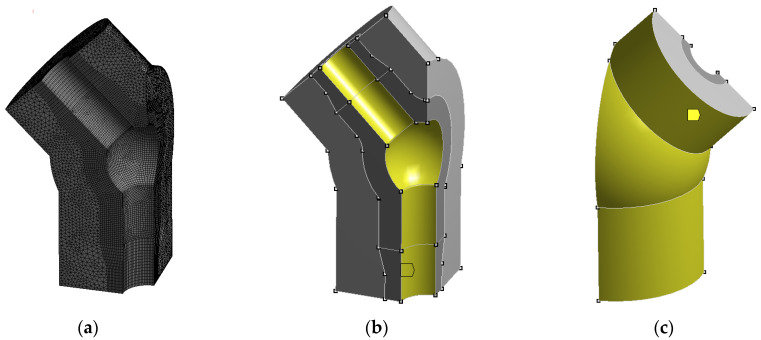
(**a**) Discrete model of the branched tee of the fresh steam pipeline made of HR6W material; (**b**) surfaces of convective heat exchange between the fresh steam and the tee; (**c**) surfaces of heat exchange between the insulation and the environment—natural convection.

The next step of the proposed methodology involves defining an appropriate discrete model of the analysed power engineering component for finite element calculations. Based on the concept of the tee, a CAD model was developed and subsequently discretized using suitable three-dimensional finite elements of the Hexa 20 and Tetra 10 types (Figure 10a). The thus-defined model of a quarter-tee with its thermal insulation consisted of 171,917 finite elements and 373,364 nodes.

Another stage of the procedure is the definition of an appropriate material model. For this purpose, in order to describe the plastic behaviour of the structural material, a suitable hardening model must be adopted.

An analysis of the hysteresis loops obtained at saturation states for specimens subjected to different levels of total strain in the HR6W material, together with the cyclic hardening curves derived from these loops, as well as information available in the literature [10,11] for other alloys used in thermal power engineering, leads to the conclusion that these characteristics describe a kinematic hardening phenomenon. This behaviour occurs between successive saturation states of the material for different levels of total strain amplitude and can be represented using a multilinear kinematic hardening (MKH) model.

Accordingly, the material model incorporates the data listed in Table 3 as well as the coordinates of the points defining the cyclic stress–strain curves shown in Figure 8.

The temperature distribution within the material volume of power engineering components has a significant influence on the stress state of their structures and, consequently, on their durability. Of particular importance are thermal shocks, defined as sudden changes in the temperature of metal surfaces in contact with the flowing working medium (e.g., steam) resulting from abrupt changes in the temperature of that medium. Such thermal shocks lead to the formation of large temperature gradients across the thickness of thick-walled components.

As a consequence of the temperature-dependent thermal expansion of the material, the geometric form of the components, and internal constraints (material resistance) or external constraints (such as the method of fixing a given power engineering component, which limits its freedom of movement), these gradients may induce deformations of sufficient magnitude to cause localised plastic deformation. Therefore, from a fatigue perspective, transient operating states of power engineering components, such as start-up and shutdown of power units, are of particular significance.

To determine the temperature distribution occurring during the operation of power engineering components, a transient thermal analysis using the finite element method should be performed [12]. In the case of the tee and steam, the primary mechanism of heat transfer between the working medium and the walls of the thick-walled vessel is convection (Figure 10b). The intensity of this process is characterised by the heat transfer coefficient α [W/(m^2^·°C)].

For the considered tee made of HR6W steel, the heat transfer coefficient was determined based on the following relationship [13]:

(2) α=0.023λdRe0.8Pr13εt

where

λ [W/(m·K)]—thermal conductivity coefficient of the fluid,

d [m]—inner diameter of the pipe,

Re [-]—Reynolds number,

Pr [-]—Prandel number,

μf, μs ([kg/(m·s)])—dynamic viscosity coefficient of the fluid for its mean temperature and wall temperature, respectively.

**Figure 11:**
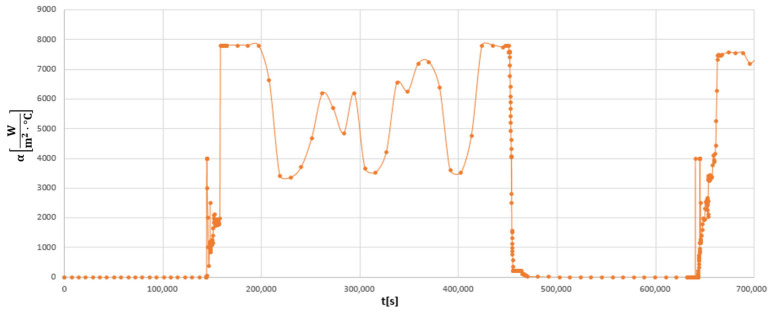
Time variation in the heat transfer coefficient α [W/(m^2^·°C)] on the inner surface of the tee made of HR6W material during the considered time period.

Based on the data presented in Figure 9 and Equation (2), the time history of the heat transfer coefficient for the analysed pipeline tee was obtained for the assumed time period (Figure 11).

Additionally, a heat transfer coefficient of α = 10 W/(m^2^·°C) was assumed for the ambient environment at an ambient temperature of 30 °C (Figure 10c).

**Figure 12:**
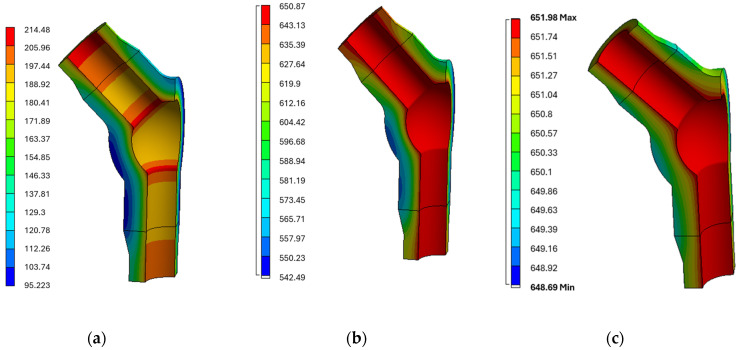
Temperature contours [°C] in the tee at (**a**) 21.448 s, (**b**) 160.200 s, and (**c**) 261.840 s during thermal analysis for the power unit start-up (**a**,**b**) and steady-state operation (**c**).

Below, exemplary temperature contours of the analysed tee obtained during transient thermal analyses are presented (Figure 12).

The next stage of the methodology for assessing the durability of power engineering components involves performing numerical strength analyses using the finite element method in order to determine the complex strain state of the component structure. To obtain results that are as close to reality as possible, these simulations must take into account the temperature field distributions, the time histories of the working-medium pressure, as well as interaction forces originating from other subsystems directly cooperating with the analysed component. The results are also strongly influenced by the external constraints (boundary conditions) applied to the numerical model, which must accurately reflect the mounting conditions of the real structure.

**Figure 13:**
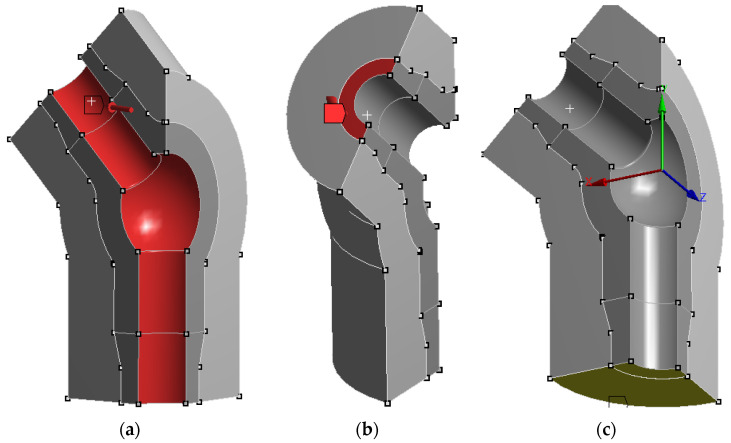
Graphical representation of the boundary conditions defined for the nonlinear strength analysis of a tee of a live steam pipeline made of HR6W material: (**a**) internal surfaces of the tee to which the live steam pressure was applied, (**b**) the surface separating the tee from the pipeline system to which a force was applied reflecting the effect of the remaining part of it on this branch, (**c**) the surface separating the tee from the pipeline system to which a restraint was applied by taking away vertical displacements from the nodes.

Figure 13a–c show the areas of the considered tee in which boundary conditions for static, nonlinear numerical calculations were assumed.

**Figure 14:**
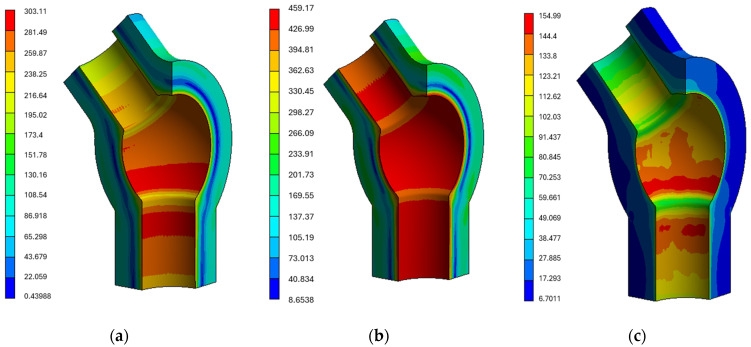
Equivalent stress contours [MPa] in the tee based on the Huber–Mises criterion at (**a**) 146.100 s, (**b**) 158.400 s during power unit start-up, and (**c**) 361.200 s during steady-state operation.

For the tee analysed within the presented methodology, the mechanical properties listed in Table 3 and a multilinear kinematic hardening model based on the cyclic stress–strain curves shown in Figure 8 were used. Selected results of the strength analyses are presented below (Figure 14a–c).

When analysing the obtained results in the form of contours of equivalent stresses according to the Huber–Mises hypothesis, attention should be paid to whether their magnitudes are sufficiently high to potentially cause plastic deformation.

**Figure 15:**
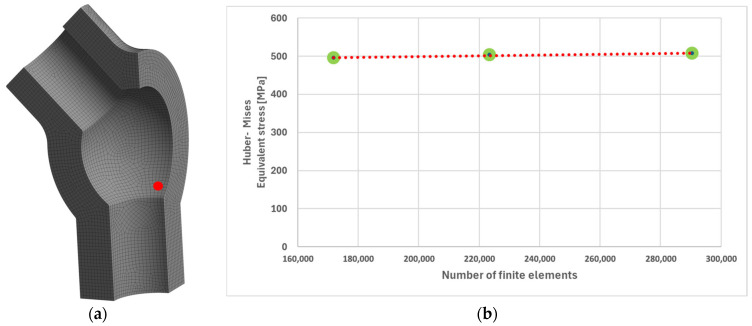
(**a**) Node number 135,678 of the tee made of HR6W material identified as the location of maximum equivalent stress values according to the Huber–Mises hypothesis; (**b**) a graph showing the independence of the results from the mesh size.

From the standpoint of both immediate strength and fatigue durability, the potentially most critical region of a power engineering component is the area of the structure where the maximum level of stress occurs, expressed by the highest value of equivalent stress according to the Huber–Mises criterion. In the case of the analysed tee, this critical location corresponds to node number 135,678, situated in the region of the lower nozzle (Figure 15a). It is worth mentioning that a mesh independence analysis was conducted. For this purpose, calculations were performed for three finite element sizes (the first mesh is the one used in the methodology, the other two are 1.3 times denser than the previous one). The results of this analysis, in the form of reduced Huber–Mises stresses in the region of their maximum values (marked in Figure 15a), for three different meshes are very similar (Figure 15b). This indicates that the adopted mesh size is correct, as increasing the mesh density does not significantly change the results.

**Figure 16:**
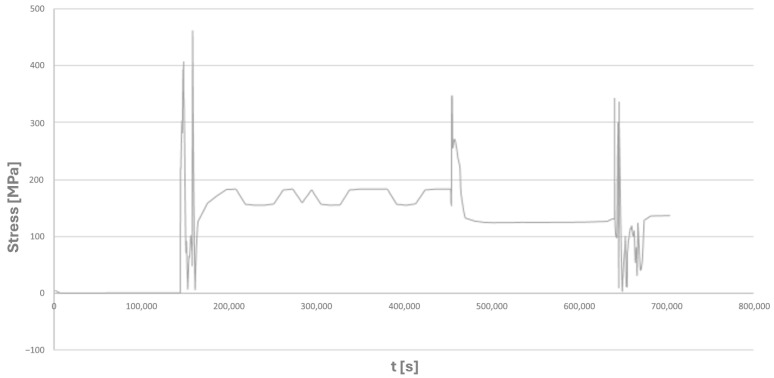
Time history of equivalent stresses according to the Huber–Mises hypothesis for node number 135,678 of the tee made of HR6W material selected for fatigue analysis (Figure 15).

**Figure 17:**
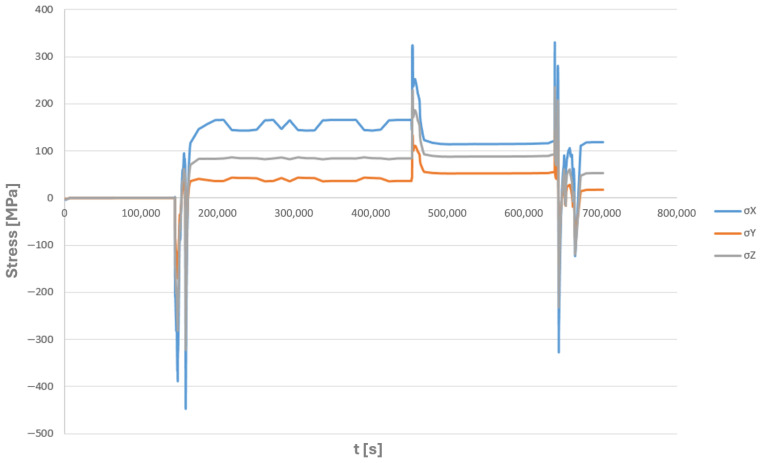
Time history of normal stresses of the assumed stress state for node number 135,678 of the tee made of HR6W material selected for fatigue analysis (Figure 15).

For node number 135,678, shown in Figure 15a, the time histories of the normal components of the complex stress state, as well as the equivalent stresses according to the Huber–Mises criterion, are presented (Figure 16 and Figure 17).

The above results allow for an unambiguous confirmation that the analysed point is subjected to the highest values of equivalent stress, reaching up to 459 MPa. Simultaneously, when considering the strength properties of the HR6W material presented in Table 3, it can be observed that during the operating cycle of the power engineering component, the equivalent stress values exceed, at certain time steps, the yield strength corresponding to the respective metal temperatures. Consequently, it can be concluded that plastic deformation may occur in the tee.

Since the multilinear kinematic hardening material model was defined using data derived from cyclic stress–strain curves, this indicates a high probability of the occurrence of thermo-mechanical fatigue in the analysed power engineering component [3].

**Figure 18:**
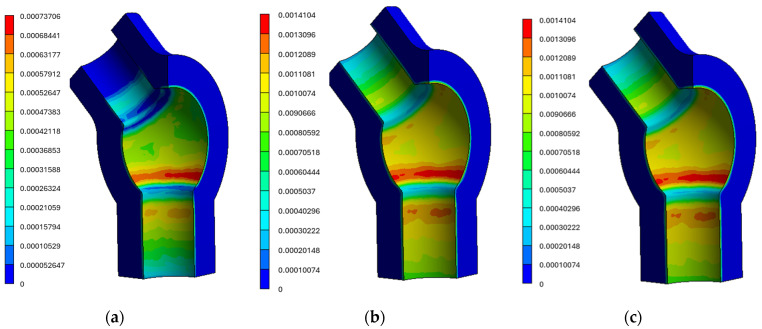
Equivalent plastic strain contours in the tee at (**a**) 148.020 s, (**b**) 158.400 s during power unit start-up, and (**c**) 361.200 s during the stress state after unit shutdown and temperature stabilisation.

Figure 18a–c below present contours of equivalent plastic strains [14].

**Figure 19:**
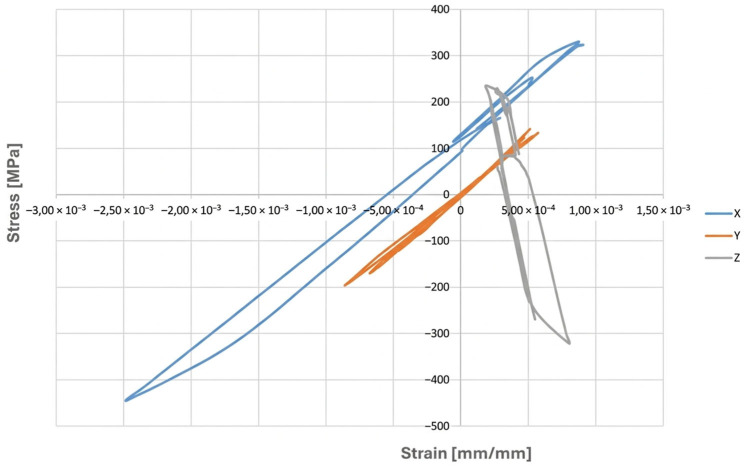
Graphical representation of the relationship between stress state components and strains at a point of the tee made of HR6W alloy, shown in Figure 15a.

In order to assess the possibility of plastic deformation occurring in each type of operating cycle represented by the finite element simulation, it is necessary to analyse, for a given point of the examined component, a graphical representation of the relationship between the stress components and their corresponding strains. Figure 19 presents such a stress–strain relationship for the forked tee made of HR6W material at the location indicated in Figure 15a.

An analysis of the above figure indicates that the stress component values in the X and Z directions exceed the yield strength of the material corresponding to the temperatures occurring during the analysed operating cycle. Moreover, the stress–strain relationships in the directions perpendicular to the X and Z axes exhibit hysteresis loop shapes, which indicates the possibility of plastic deformation occurring in each operating cycle examined using the thermo-mechanical simulations described above.

Having at one’s disposal the results of low-cycle fatigue tests conducted at elevated temperatures as well as the numerical analysis results for power engineering components, the question arises as to how fatigue durability should be evaluated. In the generally available literature, and particularly in normative documents such as EN 12952-4:2011 [15] and ASME Code Cases—Boilers and Pressure Vessels [16], an approach is adopted that involves identifying load cycles within the load history using the rainflow counting method, followed by counting cycles with the same stress range.

Another approach, described, for example, in Refs. [17,18], involves determining various criterion quantities related to the number of cycles to failure. Since the results of low-cycle fatigue tests at elevated temperatures and, indirectly, thermo-mechanical fatigue tests include hysteresis loops, certain geometric parameters of these loops can be used to define such criterion functions. Of particular importance in this context are the loops corresponding to the saturation state, which, under combined thermal loading (temperature field) and mechanical loading (elongation induced by the testing machine), do not change their position, shape, or size.

It is precisely the geometric parameters determined for these saturation hysteresis loops that are correlated with the number of cycles to failure of specimens tested at a given total strain range and test temperature.

In the scientific literature, criterion values determined on the basis of hysteresis loops corresponding to the saturation state of specimens can be found, such as the Coffin and Ostergren parameters, which are defined by the following relationships:

(3) $$C\left(N_{f}\right)=∆\varepsilon_{p}\left({∆\varepsilon }_{c},T\right)$$

(4) $$O\left(N_{f}\right)=∆\varepsilon_{p}\left({∆\varepsilon }_{c},T\right)\cdot \sigma_{{an}_{max}}$$

where

$N_{f}$—the number of cycles to failure determined from low-cycle fatigue tests conducted at an elevated temperature for a specimen uniformly heated throughout its entire volume to a temperature T (°C), subjected to fully reversed cyclic loading with a stress ratio of R = −1 and a total strain amplitude of ∆ε_c_.

$∆\varepsilon_{p}(∆\varepsilon_{c},T$)—the plastic strain range obtained for a specimen subjected to a low-cycle fatigue test with a total strain range ∆ε_c_ at a temperature of T degrees Celsius.

$\sigma_{{an}_{max}}$—the stress amplitude (stress value) for the specimen in the saturation state.

$C(N_{f})$—the Coffin parameter as a criterion function used for estimating the fatigue life of components subjected to low-cycle fatigue at elevated temperatures.

$O\left(N_{f}\right)$—The Ostergren parameter, a criterion function used to estimate the fatigue life of components subjected to low-cycle fatigue at elevated temperatures.

**Figure 20:**
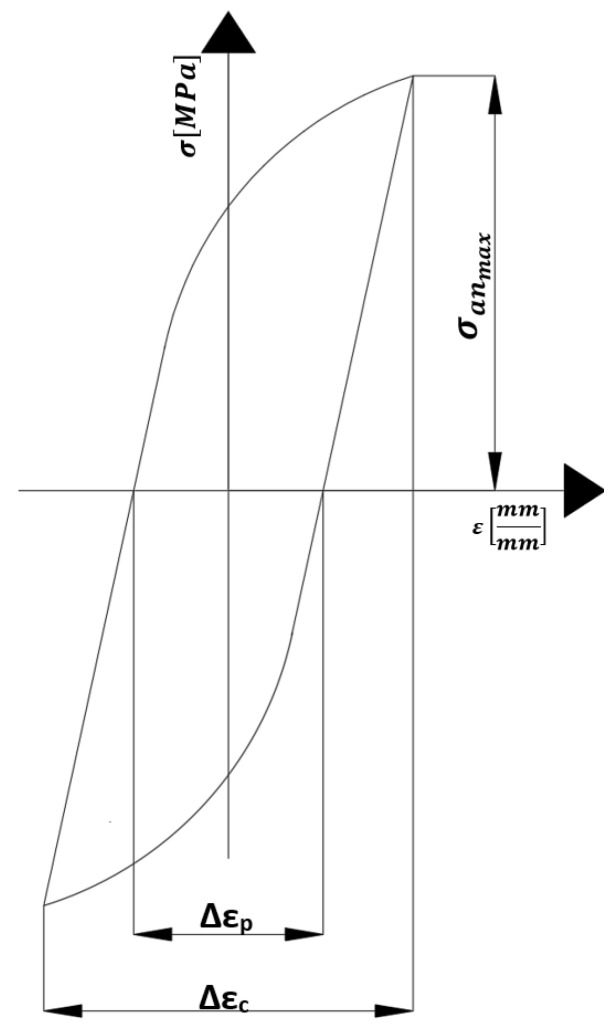
Assumptions for determining the Coffin and Ostergren parameters based on the hysteresis loop corresponding to the saturation state of specimens subjected to low-cycle fatigue tests.

Figure 20 illustrates the assumptions adopted for determining the Coffin and Ostergren parameters corresponding to the saturation state of the specimens, based on hysteresis loops obtained from low-cycle fatigue tests carried out up to the saturation condition.

**Figure 21:**
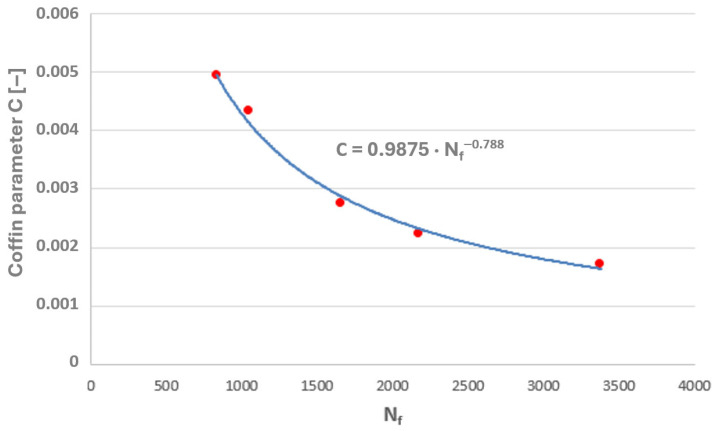
Relationship between the Coffin parameter and the number of cycles to failure (N_f_) for the HR6W alloy at 650 °C.

**Figure 22:**
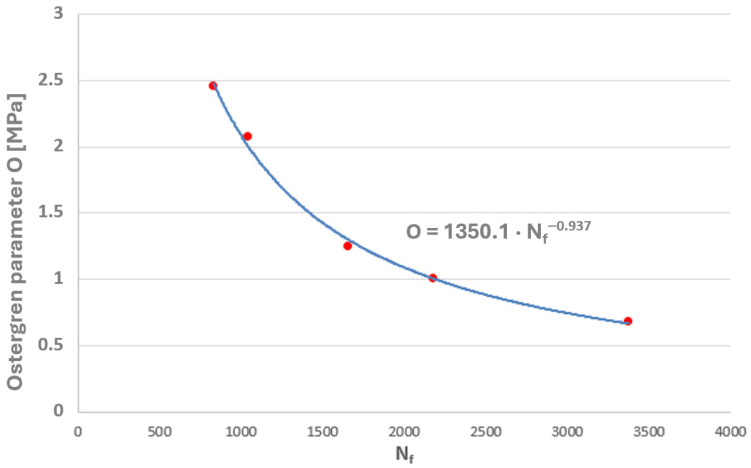
The relationship between the Ostergren parameter and the number of cycles to failure (N_f_) for the HR6W alloy at 650 °C.

Taking into account the results of the authors’ own low-cycle fatigue tests conducted at 650 °C, plots of the Coffin and Ostergren parameters as functions of the number of cycles to failure were prepared (Figure 21 and Figure 22).

**Figure 23:**
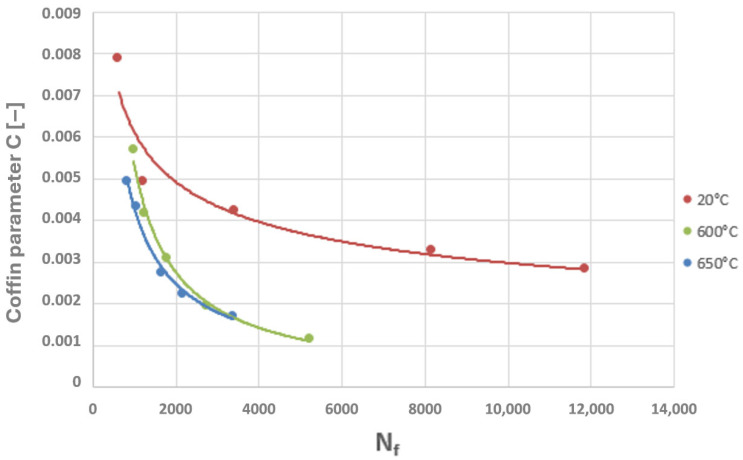
Summary of the relationship between the Coffin parameter and the number of cycles to failure (N_f_) for the HR6W alloy at various temperatures.

**Figure 24:**
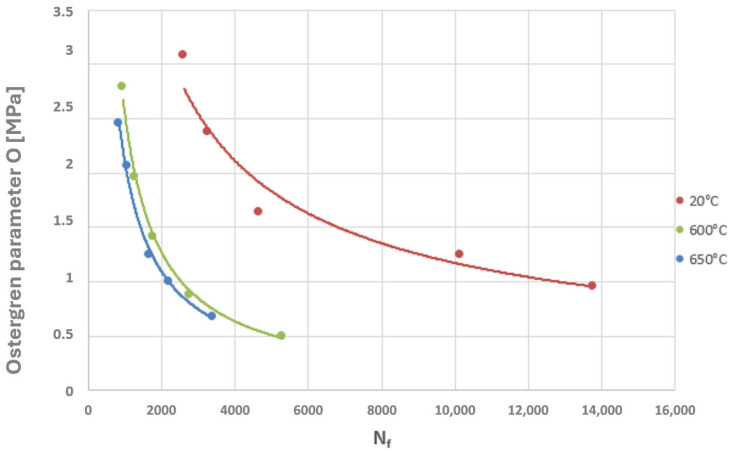
Summary of the relationship between the Ostergren parameter and the number of cycles to failure (N_f_) for the HR6W alloy at various temperatures.

A power-law character of the distribution of these parameter values as a function of the number of cycles to failure can be observed, which corresponds to the nature of the graphical representation of the plastic strain component of the Manson–Coffin equation. The characteristics presented in Figure 23 and Figure 24 compare the values of the Coffin and Ostergren parameters for several temperature levels, based on data obtained from the authors’ own experiments, as well as from the literature.

Considering the two diagrams above, it can be observed that an increase in temperature leads to a reduction in the fatigue life of the HR6W alloy, manifested by a decrease in the number of cycles to failure accompanied by a simultaneous reduction in the values of the Coffin and Ostergren parameters.

At the same time, it is necessary to return to the initial assumption concerning the performance of low-cycle fatigue experimental tests at a constant, elevated temperature in order to describe the phenomenon of thermo-mechanical fatigue. Specifically, it was assumed that by conducting low-cycle fatigue tests at an elevated, constant temperature for a selected range of total strain amplitudes, it is possible to reflect the influence on the material durability of time-dependent thermal and mechanical strains characteristic of thermo-mechanical fatigue. A similar assumption can be made when determining the fatigue life of the alloy based on the relationships between the Coffin and Ostergren parameters and the number of cycles to failure. This implies that, in order to assess the loss of durability for a specific type of operating cycle of a power-generation component in which thermo-mechanical fatigue occurs, the appropriate relationships of the above criterion functions, determined from low-cycle fatigue tests under isothermal conditions, can be applied. Confirmation of this hypothesis is provided in publications [19,20].

In that study, a high degree of agreement was demonstrated between the results obtained from low-cycle fatigue tests and thermo-mechanical fatigue tests for X20CrMoV12-1 steel with respect to the values of the criterion function proposed by the authors, namely the coefficient P, which is closely related to the quantities $C\left(N_{f}\right)$ and $O\left(N_{f}\right)$, and is described by the following relationship:

(5) $$P\left(N_{f}\right)=∆\varepsilon_{p}\left({∆\varepsilon }_{c},T\right)\cdot \sigma_{{an}_{max}}\cdot T_{max}=O\left(N_{f}\right){\cdot T}_{max}=C\left(N_{f}\right)\cdot \sigma_{{an}_{max}}\cdot T_{max}$$

where $T_{max}$—the maximum temperature value within the fatigue cycle.

In view of the above, it may be assumed that, also in the case of the Ostergren and Coffin parameters, only a minor difference would occur between the two types of fatigue for the X20CrMoV12-1 alloy.

**Figure 25:**
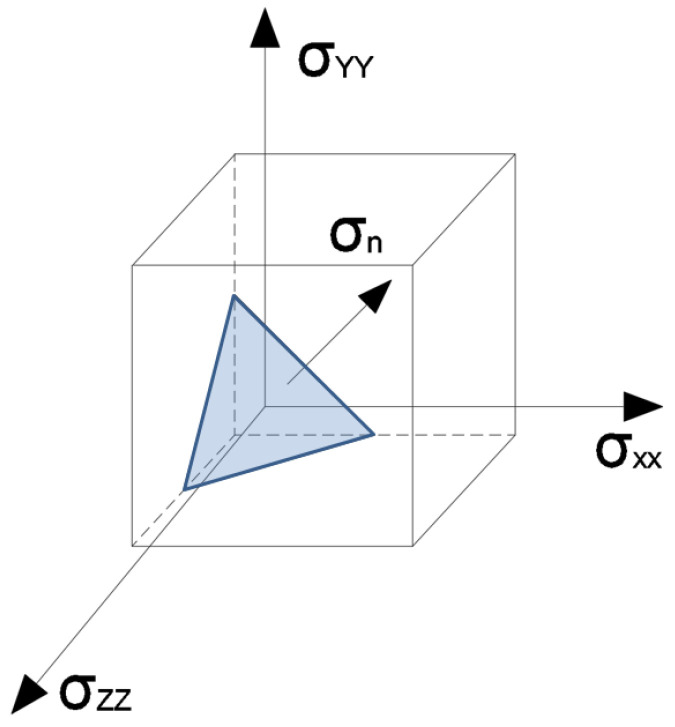
Normal stress vector for determining the critical plane.

The values of the criterion functions obtained from low-cycle fatigue tests at an elevated temperature should be compared with those determined for actual power-generation components using the finite element method. In order to assess fatigue life under a complex stress state derived from a thermo-mechanical analysis of a power component, an approach based on the determination of the critical plane may be employed, as described in detail in publications [3,21]. This plane represents the region in which the criterion values, i.e., the Coffin and Ostergren parameters determined from hysteresis loops induced by the normal stresses $\sigma_{n}$ acting on this plane, reach their maximum values (Figure 25) [22,23].

**Figure 26:**
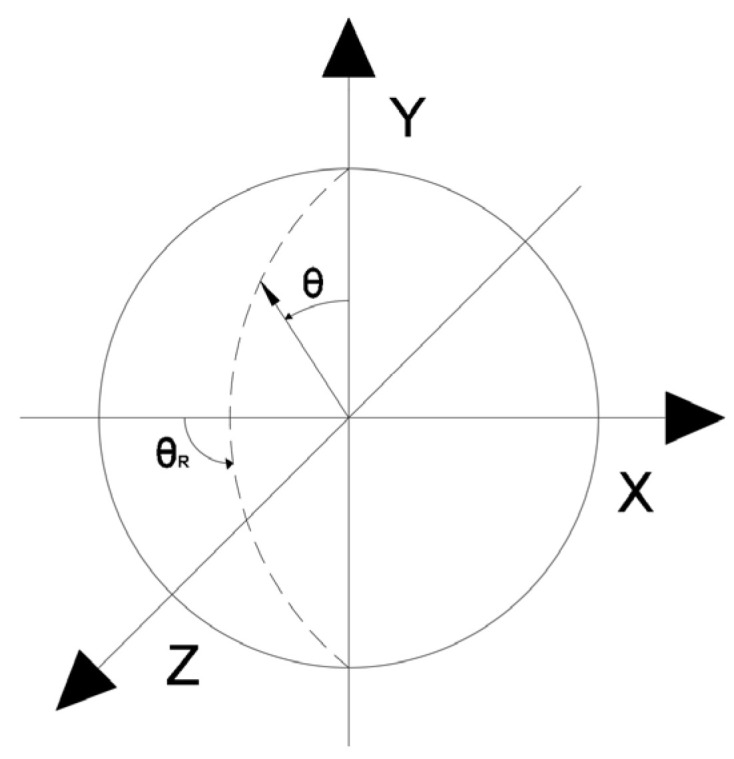
Graphical representation of the definitions of angles θ and θR determining the orientation of potential critical planes.

For a structural point of a power-generation component analysed in terms of fatigue durability, the procedure for determining life reduction using the critical plane method can be divided into several stages. The first stage involves identifying potential critical planes, defined in three-dimensional space by the angles θ and θR, as illustrated in Figure 26 [24].

The coefficients necessary for determining the critical planes can be defined according to the relationships given below:

(6) $$n_{Y}=-sinsin\theta \cdot coscos\theta_{R}$$

(7) $$n_{X}=sinsin\theta \cdot sinsin\theta_{R}$$

(8) $$n_{Z}=coscos\theta$$

where θϵ⟨0°,180°⟩ i $\theta_{R}\epsilon \langle 0{}^{\circ},180{}^{\circ}\rangle$.

For the given ranges of angles, the parameter values expressed by Equations (6)–(8) allow tracing the full set of potentially critical planes due to the properties of trigonometric functions (symmetry of their values). Subsequently, using the above parameters, for each pair of angles θ and θR (defining the plane), the normal stresses and strains on the identified plane must be determined as described by the following equations:

(9) $$\sigma_{n}=\sigma_{X}\cdot n_{X}^{2}+\sigma_{Y}\cdot n_{Y}^{2}+\sigma_{Z}\cdot n_{Z}^{2}+2\cdot \tau_{XY}\cdot n_{X}\cdot n_{Y}\;+2\cdot \tau_{YZ}\cdot n_{Y}\cdot n_{Z}+2\cdot \tau_{XZ}\cdot n_{X}\cdot n_{Z}$$

(10) $$\varepsilon_{n}=\varepsilon_{X}\cdot n_{X}^{2}+\varepsilon_{Y}\cdot n_{Y}^{2}+\varepsilon_{Z}\cdot n_{Z}^{2}+\gamma_{XY}\cdot n_{X}\cdot n_{Y}+\gamma_{YZ}\cdot n_{Y}\cdot n_{Z}+\gamma_{XZ}\cdot n_{X}\cdot n_{Z}$$

where σ_X_, σ_Y_, σ_Z_, τ_XY_, τ_YZ_, τ_XZ_, $\varepsilon_{X}$, $\varepsilon_{y}$, $\varepsilon_{z}$, γ_XY_, γ_YZ_, γ_XZ_—components of the combined stress and strain state at the point of the power device where fatigue life is determined [25,26].

Thus, if the results of numerical analyses in the form of stress and strain state components for specific points of power equipment are functions of computational time, then based on points with coordinates ($\varepsilon_{n}$, $\sigma_{n}$), hysteresis loops characterising the cyclic behaviour of the structural material can be constructed. By varying the analysed plane through different values of angles ϴ and ϴR, a new set of points with coordinates ($\varepsilon_{n}$, $\sigma_{n}$) is obtained, and consequently, a new hysteresis loop is generated. Among the resulting $\varepsilon_{n}$ − $\sigma_{n}$ plots, those for which the Coffin parameter $C(N_{f})$ and the Ostergren parameter $O(N_{f})$ reach their maximum values must be identified. The pairs of angles ϴ and ϴR corresponding to these maxima define the critical planes. Next, the maximum values of the parameters C and O, determined indirectly based on the results of numerical calculations, should be related to the dependencies of these quantities as functions of the number of cycles to failure $N_{f}$, established during laboratory tests on specimens (dependencies of the same type as those presented in Figure 21, Figure 22, Figure 23 and Figure 24) for the temperature equal to the highest temperature occurring in the fatigue cycle. Since experimental test results are usually not available at the exact highest temperature occurring during the analysed period, it is advisable to consider the dependency determined for a temperature as close as possible to, and simultaneously higher than, the temperature used in the calculations. This approach is justified by the fact that the fatigue life of metals decreases with increasing temperature, as shown in the graphs in Figure 23 and Figure 24. Such an approach results in assessing the fatigue life of the power equipment at a conservative (lower than actual) level. Therefore, based on the calculation results, a safe fatigue life limit for the fresh steam pipeline tee has been determined [27,28].

**Figure 27:**
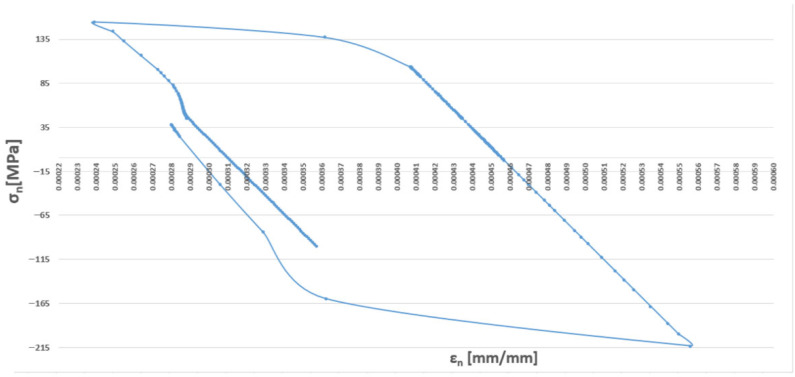
Hysteresis loop obtained from numerical calculations of the analysed tee for node 135,678 selected for fatigue analysis, in the plane defined by angles ϴ = 90° and ϴR = 165°.

An example hysteresis loop plotted in the $\varepsilon_{n}$ − $\sigma_{n}$ coordinate system for the node selected to determine the fatigue life of the analysed tee (Figure 15a) at different potential planes defined by angles ϴ and ϴR is shown in Figure 27.

Analysing the cyclic strain curves (Figure 8), hysteresis loops in Figure 19, and the Coffin and Ostergren function plots (Figure 23 and Figure 24), it can be concluded that the HR6W alloy exhibits a tendency for a significant increase in strength properties (hardening) accompanied by a reduction in ductility, as measured by the hysteresis loop width. This, in turn, explains the high fatigue durability of this steel despite its tendency to develop high thermal stresses caused by thermal shocks [29].

**Figure 28:**
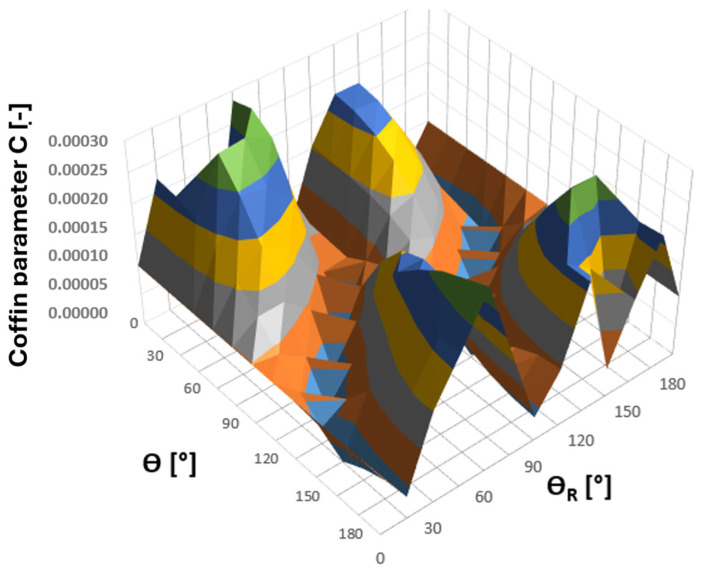
Values of the Coffin parameter C as a function of angles ϴ and ϴ_R_ determined for node 135,678 of the tee made of HR6W material.

**Figure 29:**
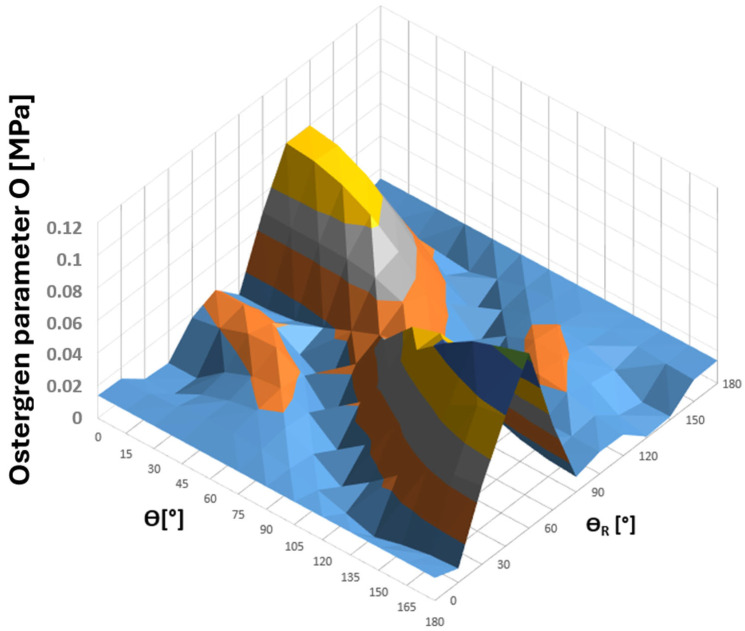
Values of the Ostergren parameter O [MPa] as a function of angles ϴ and ϴ_R_ for node 135,678 of the tee made of HR6W material.

For the tee point selected for fatigue analysis according to Figure 26, a survey of planes was conducted with respect to the values of the Coffin and Ostergren parameters obtained from the analysis of the resulting hysteresis loops in the $\varepsilon_{n}$ − $\sigma_{n}$ coordinate system. The search was performed over the angular ranges of ϴ and ϴ_R_ equal to ⟨0°,180°⟩, with increments of 15°. The results of these calculations, in the form of 3D plots showing the parameter values as functions of angles ϴ and ϴ_R_, are presented in Figure 28 and Figure 29 [30,31].

**Figure 30:**
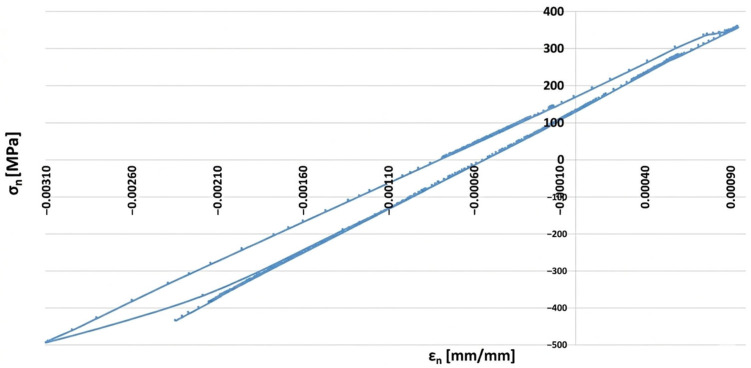
Hysteresis loop obtained from numerical calculations of the tee made of HR6W material for node 135,678 in the critical plane defined by angles ϴ = 180° and ϴ_R_ = 60°.

Analysing the above plots, it can be observed that they exhibit a continuous nature, indicating that the Coffin and Ostergren criterion functions were correctly determined based on the components of the combined stress and strain state at node 135,678 of the tee (Figure 30). At the same time, regions of local extrema of these quantities are clearly visible, illustrating their variability across all possible planes. This, in turn, highlights the necessity to identify the critical plane for which the chosen criterion parameters reach their maximum values. Considering the maximum normal stress criterion [21,30], the combined strain state (and consequently the stress) determined in this plane for a precisely defined region of the structure is the most unfavourable from the fatigue life perspective.

Considering the above results, both the Coffin and Ostergren parameters reach their maximum values for the same plane defined by the angle pair ϴ = 180° and ϴ_R_ = 60°, with C_max_ = 0.000295841 and O_max_ = 0.107262268, respectively. The hysteresis loop corresponding to the identified critical plane is presented in Figure 30 [31,32].

**Figure 31:**
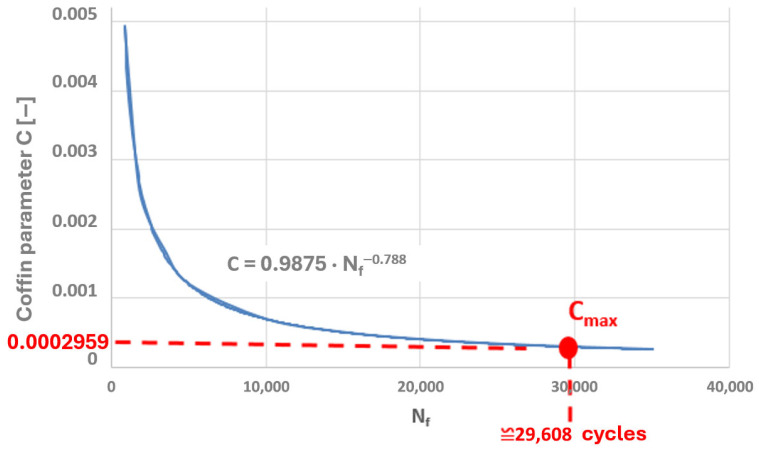
Determination of the number of cycles to failure by plotting the maximum value of the Coffin parameter C_max_ on the graph of this parameter as a function of the number of cycles to failure, established based on low-cycle fatigue laboratory tests conducted at 650 °C.

**Figure 32:**
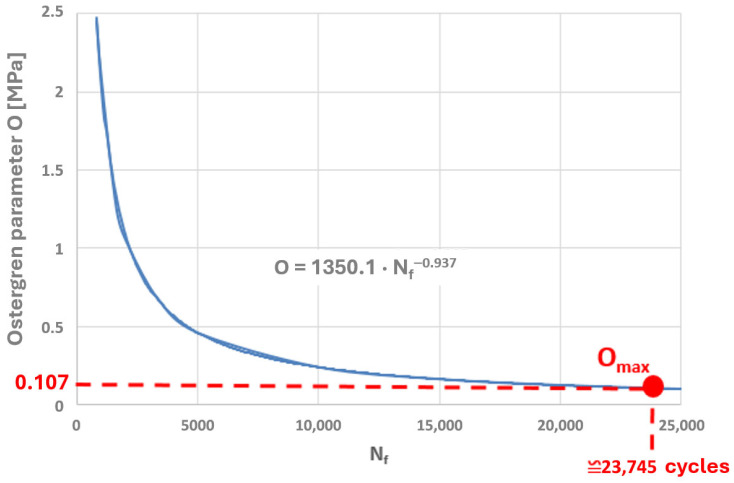
Determination of the number of cycles to failure by plotting the maximum value of the Ostergren parameter O_max_ on the graph of this parameter as a function of the number of cycles to failure, established based on low-cycle fatigue laboratory tests conducted at 650 °C.

The maximum values of both parameters were plotted on the graphs of these quantities, determined based on laboratory tests on specimens at 650 °C (where the highest temperature obtained on the tee surface during thermal calculations of its operating cycle was approximately 652 °C). The corresponding numbers of cycles to failure were thus determined (Figure 31 and Figure 32).

Consequently, the determined numbers of cycles to failure for the Coffin and Ostergren parameters are 29,608 and 23,745, respectively. From the perspective of the tee’s operational safety, the lower value, i.e., 23,745 (corresponding to the Ostergren parameter), should be adopted for fatigue life calculations. Thus, the unit fatigue damage of the analysed tee made of HR6W material, part of the fresh steam pipeline, caused by a single operation cycle characterised by a cold start-up and normal shutdown of the unit, would be equal to [33,34]:

(11) $$∆D_{U}=\frac{n}{N}=\frac{1}{23,745}$$

## 4. Conclusions

Based on the content of the above article, the following conclusions can be drawn:

1. The present publication outlines a methodology for assessing the fatigue life of power equipment, which can be applied both during the design process of new power devices and for determining fatigue damage resulting from thermo-mechanical fatigue in already operated installations.
2. The fundamental assumption made in the article is that the evaluation of fatigue life reduction in power equipment operating under thermo-mechanical fatigue conditions can be conducted based on the cyclic properties of materials obtained from low-cycle fatigue tests at elevated temperatures. This hypothesis is grounded in the analysis of results presented in publication [1], where the fatigue lives of specimens made of the material X20CrMoV12-1 subjected to low-cycle fatigue testing at elevated temperatures and thermo-mechanical fatigue tests with similar ranges of total strain $∆\varepsilon_{c}$ were compared. The calculation results confirmed a high correlation in the values of the criterion function describing the fatigue life of the specimens as a function of the number of cycles to failure for both types of fatigue. Consequently, by analogy, it was assumed that the fatigue lives for both phenomena, at the same number of cycles to failure, are comparable.
3. This method allows for the extrapolation or interpolation of data obtained from the literature and laboratory equipment regarding the temperature of metal alloys for the initial values, in order to obtain the emission power at intermediate temperatures. This, in turn, influences the results obtained.
4. The proposed methodology allows for the optimisation of the procedure for changing the operating parameters of power units during operation, with the aim of ensuring greater fatigue life.
5. The above methodology enables the selection of appropriate construction materials for power equipment to ensure adequate fatigue life.

## Figures and Tables

**Table 1:** Values of the coefficients k and n of the cyclic hardening curves for the HR6W alloy at different temperatures [2,9].

t [°C]	k [MPa]	n [-]
20	923.95	0.1567
600	1402.5	0.2042
650	2275.4	0.2943

**Table 2:** Coefficients k and n of cyclic hardening curves for the HR6W alloy determined at different temperatures.

t [°C]	E [MPa]	k [MPa]	n [-]
20	192,000	923.95	0.1567
100	188,000	984.9	0.1652
200	182,000	1110.5	0.1816
300	175,000	1277.3	0.2014
400	169,000	1440.2	0.219
500	164,000	1591.7	0.2341
600	156,000	1402.5	0.2042
650	151,000	2275.4	0.2943

**Table 3:** Thermo-mechanical properties of the HR6W alloy determined based on reference [1].

	Temperature [°C]							
	20	100	200	300	400	500	600	650
Young’s modulus E [MPA]	192,000	188,000	182,000	175,000	169,000	164,000	156,000	151,000
Yield strength Re0.2 [MPa]	235	215	185	170	160	155	150	148
Thermal conductivity coefficient λ$\left[\frac{W}{m\times K}\right]$	10.8	12.7	15.1	17.4	19.9	23.4	26.1	29.4
Thermal expansion coefficient β$\left[\frac{1}{K}\right]$	1.2 × 10^−5^	1.36 × 10^−5^	1.42 × 10^−5^	1.45 × 10^−5^	1.49 × 10^−5^	1.51 × 10^−5^	1.52 × 10^−5^	1.536 × 10^−5^
Specific heat Cp $\left[\frac{J}{kg\times K}\right]$	442	482	521	549	578	636	665	684.7

## Data Availability

The original contributions presented in this study are included in the article. Further inquiries can be directed to the corresponding author.
